# Exploration of marine natural resources in Indonesia and development of efficient strategies for the production of microbial halogenated metabolites

**DOI:** 10.1007/s11418-021-01557-3

**Published:** 2021-08-20

**Authors:** Hiroyuki Yamazaki

**Affiliations:** Faculty of Pharmaceutical Sciences, Tohoku Medical and Pharmaceutical University, Sendai, 981-8558 Japan

**Keywords:** MONOTORI, Indonesia, Marine invertebrates, Fungi, Organohalogens, Biological activities

## Abstract

Nature is a prolific source of organic products with diverse scaffolds and biological activities. The process of natural product discovery has gradually become more challenging, and advances in novel strategic approaches are essential to evolve natural product chemistry. Our focus has been on surveying untouched marine resources and fermentation to enhance microbial productive performance. The first topic is the screening of marine natural products isolated from Indonesian marine organisms for new types of bioactive compounds, such as antineoplastics, antimycobacterium substances, and inhibitors of protein tyrosine phosphatase 1B, sterol *O*-acyl-transferase, and bone morphogenetic protein-induced osteoblastic differentiation. The unique biological properties of marine organohalides are discussed herein and attempts to efficiently produce fungal halogenated metabolites are documented. This review presents an overview of our recent work accomplishments based on the MONOTORI study.

**Graphic abstract:**

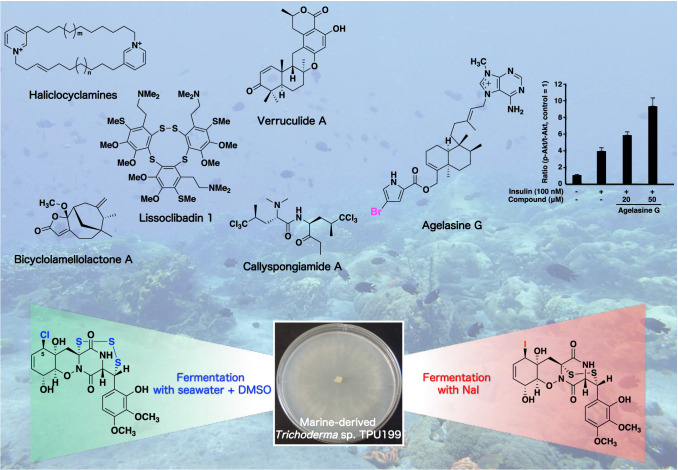

## Introduction

The search for bioactive natural products from plants and microorganisms followed by marine invertebrates is called “MONOTORI”, and has made a significant contribution to the discovery and development of various pharmaceutical applications for global health and care [1, 2].

Important research on two natural products won the 2015 Nobel Prize in Physiology or Medicine [3]. One award winner was artemisinin, which was isolated from the Chinese folk medicinal plant *Artemisia annua* and is very effective against malaria [4]. Another award winner was avermectin produced by soil-derived *Streptomyces avermitilis*, and its dihydro-derivative, ivermectin, is clinically used to treat roundworm parasites [5]. Over the past few decades, natural product chemists have shifted their focus to bioresources with access difficulties, and marine organisms have been in the spotlight as the next suppliers of highly diverse natural products in addition to terrestrial organisms [6–8]. Some marine substances have been in clinical trials for the treatment of cancers, and cytarabine (a pyrimidine nucleoside), trabectedin (ET743), eribulin (a synthetic derivative of halichondrin B), brentuximab vedotin (an antibody drug conjugate of monomethylauristatin E), and plitidepsin (dehydrodidemnin B) have already been approved as anticancer agents [9]. These natural compounds introduced are “a splendid gift from the Earth” [10], and this research area will continuously provide exciting outcomes.

I and my collaborators have also been investigating MONOTORI studies aimed at marine organisms and microorganisms mainly collected in tropical and subtropical regions. We herein review the following findings of our recent studies: (i) bioactive compounds from Indonesian marine invertebrates and microorganisms; (ii) the unique biological properties of the marine organohalogen; and (iii) the efficient production of microbial halogenated metabolites.

## The search for bioactive substances from Indonesian marine organisms

The Republic of Indonesia (commonly called Indonesia) is one of the Southeast Asian countries surrounding the Indian and Pacific oceans, and is the largest island country in the world, consisting of more than 10,000 islands, primarily Sumatra, Java, and Sulawesi.

**Fig. 1:**
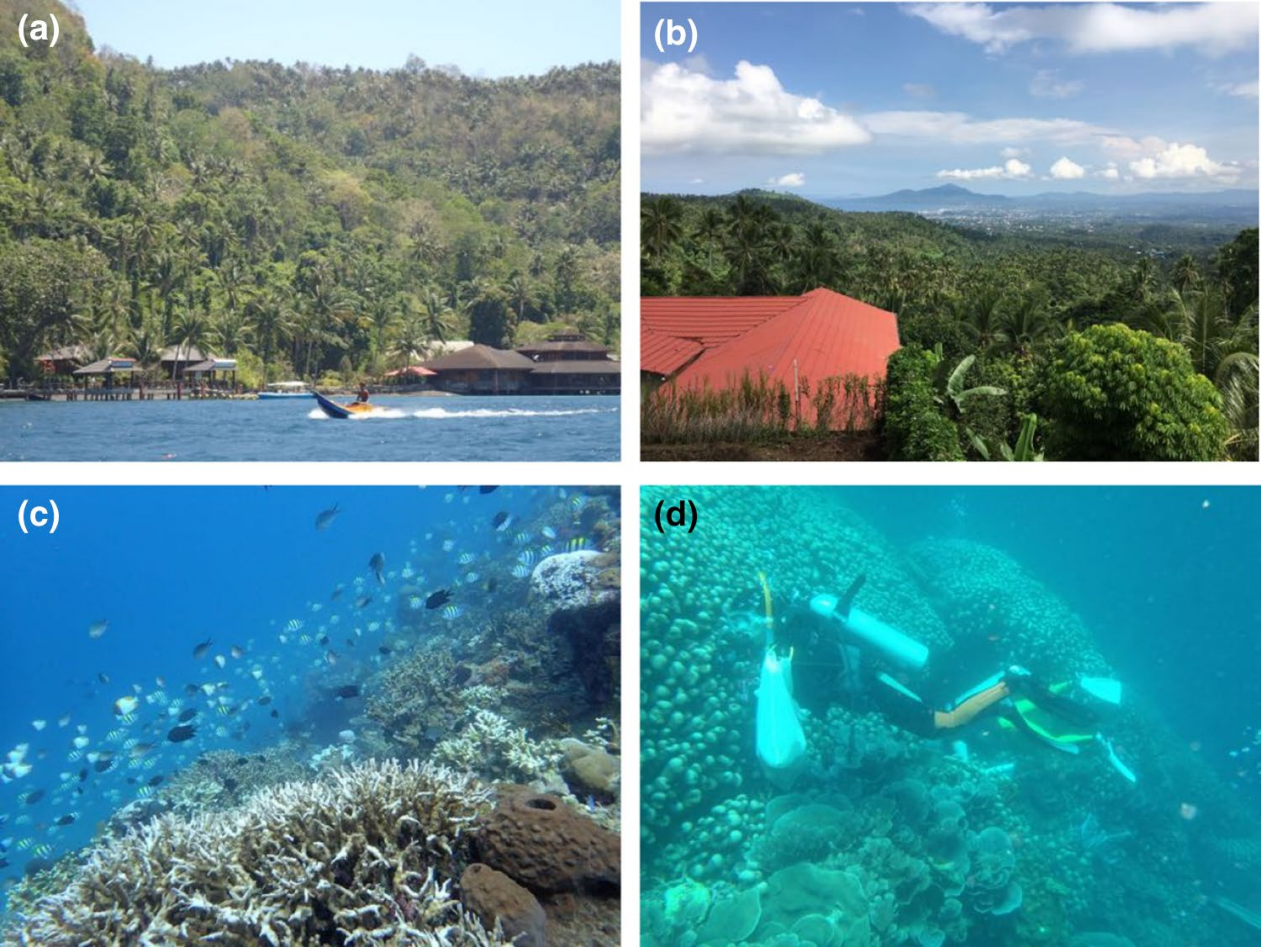
**a** and **b** Rich natural environments in North Sulawesi; **c** coral reefs in the sea of North Sulawesi; **d** sampling of marine organisms by scuba diving (the diver in the picture is the author)

Our research group has been collaborating with Sam Ratulangi University (UNSRAT: Universitas Sam Ratulangi in Indonesia) located in Manado, North Sulawesi in Indonesia. North Sulawesi is an archipelagic area on the Minahasa Peninsula of Sulawesi Island that maintains numerous natural resources (Fig. 1a, b). Abundant coral reefs are well preserved over adjacent oceans in which widely diverse native marine organisms live without invasion by foreign species (Fig. 1c). Therefore, we conducted field work in the ocean of North Sulawesi to collect marine invertebrates (ascidians and marine sponges) and marine-derived microorganisms by scuba diving (Fig. 1d), and investigate chemical constituents for their structural and biological characteristics using various bioassay screening techniques. We herein summarize the novel bioactive compounds (containing structurally known compounds) found during our search of marine bioresources in North Sulawesi that exhibit anticancer, antimycobacterial, antidiabetes, antidyslipidemia, and antiosteoblastogenic activities.

## Anticancer compounds

**Fig. 2:**
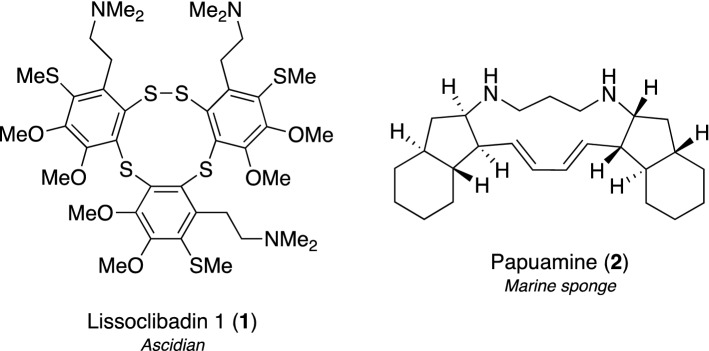
Structures of lissoclibadin 1 (**1**) and papuamine (**2**) as anticancer compounds

Some marine natural products have been approved as anticancer agents. Based on this background, we initially attempted to identify cytotoxic compounds and discovered two rare types of alkaloids with interesting features, lissoclibadin 1 (**1**) and papuamine (**2**), as shown in Fig. 2 [11–14].

Lissoclibadins, novel dopamine-derived polysulfur alkaloids, were initially isolated from the Indonesian colonial ascidian *Lissoclinum* cf. *badium* by our research group, and 14 related congeners, lissoclibadins 1–14, were isolated by further efforts [15–20]. Among them, lissoclibadin 1 (**1**), a trimeric derivative with a ten-membered polysulfur ring (Fig. 2), exerted the most potent growth-inhibitory effects against four human solid cancer cell lines, HCT-15 (colon adenocarcinoma), HeLa-S3 (cervix adenocarcinoma), MCF-7 (breast adenocarcinoma), and NCI-H28 (mesothelioma), in in vitro cytotoxicity assays. A flow cytometric study using HCT-15 cells stained by fluorescein isothiocyanate-conjugated Annexin V and propidium iodide in the presence or absence of caspase inhibitors (z-VAD-fmk, z-IETD-fmk, and z-LEHD-fmk) confirmed that compound **1** promoted the induction of apoptosis, which was attributed to the intrinsic pathway of the caspase cascade, namely, the mitochondrial cytochrome *c*-dependent activation of caspase-9 and caspase-3 in HCT-15 cells. Compound **1** suppressed in vivo tumor growth in nude mice carrying HCT-15 cells by approximately 60% on day 28 at 25 mg/kg per day without any severe side effects or body weight changes.

Papuamine (**2**), an unusual pentacyclic diamine alkaloid, was originally isolated as a fungicide against *Trichophyton mentagrophytes* from the Papua New Guinean marine sponge *Haliclona* sp. [21]. We also discovered the same alkaloid **2** in Indonesian *Haliclona* sp. (Fig. 2) and its potent cytotoxicity against the human solid cancer cell lines, MCF-7, HCT-15, Caco-2 (colon adenocarcinoma), and LNCap (prostate adenocarcinoma) [12]. Further biochemical experiments on the cytotoxic mechanism of **2** against MCF-7 cells revealed autophagosome vesicular formation by the detection of LC3, a typical marker of mammalian autophagy, and the release of cytochrome *c* coincided with the activation of c-Jun N-terminal kinase (JNK), indicating that compound **2** induces an earlier onset of autophagy, followed by a reduction in cell survival through mitochondrial damage and the activation of JNK in MCF-7 cells [13]. Additionally, in our examination to evaluate synergistic effects with doxorubicin (DOX), a major chemotherapeutic reagent that activates JNK, the combination of **2** and DOX exhibited stronger cytotoxicity against MCF-7 cells, which did not involve changes in the cellular accumulation of DOX and appeared to reflect the additional activation of JNK phosphorylation [14].

## Antimycobacterial substances

Infectious diseases are the greatest public health threat worldwide; however, since the discovery of penicillin in 1928, several antibiotics have historically overcome epidemics [2, 4, 5, 22]. In other words, natural product chemistry has made progress to combat infections. Therefore, researchers have continually explored new antiinfective candidates [23, 24]. We also investigated novel antiinfective leads against several pathogens [25–32].

*Mycobacterium tuberculosis* causes tuberculosis (TB), which is one of the three major infectious diseases, including human immunodeficiency virus (HIV) and malaria, worldwide [33]. The treatment of TB is challenging due to the prevalence of multidrug resistance, the limited number of anti-TB agents, and long-term administration; therefore, the exploitation of new anti-TB drugs with novel modes of action globally is needed [34, 35]. Experiments using *M*. *tuberculosis* are tightly restricted by the requirement of a biosafety level 3 facility and time-consuming assays because of the pathogenicity and slow growth of *M*. *tuberculosis*, respectively. Our project to search for antimycobacterium activity has applied non-pathogenic and fast-growing *M*. *smegmatis*, the susceptibility of which to anti-TB drugs is consistent with that of *M*. *tuberculosis* [36], as an alternative test strain to detect antituberculous activity [37–42].

**Fig. 3:**
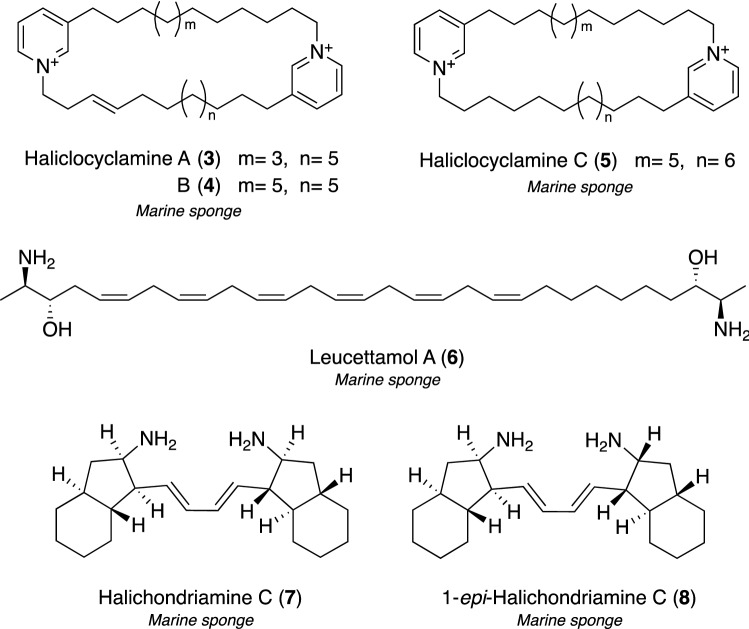
Structures of marine antimycobacterial substances **3**–**8**

In this screening, we found that an ethanol (EtOH) extract of the Indonesian marine sponge *Haliclona* sp. exhibited antimycobacterial activity against *M*. *smegmatis* [37]. ODS and HPLC separation according to bioassays gave haliclocyclamines A–C (**3**–**5**) and five known congeners, cyclostellettamines A–C, E, and F [43]. The structures of **3**–**5** were elucidated as new dimeric 3-alkyl pyridinium alkaloids based on their NMR spectra in combination with ESI–MS/MS analyses (Fig. 3). The inhibitory efficacies of **3**–**5** against the growth of *M. smegmatis* were assessed using the paper disc method [44]. Compound **3** exhibited the most potent activity, in a dose-dependent manner, with an inhibition zone of 17 mm at 10 μg/disc. Since anti-*M. tuberculosis* activity by cyclostellettamines, compounds related to **3**–**5**, has been demonstrated [45], compounds **3**–**5** are also expected to be active against *M. tuberculosis*.

Leucettamol A (**6**), a known dimeric sphingolipid (Fig. 3), was isolated as an anti-*M. smegmatis* component from the Indonesian marine sponge *Agelas* sp. [38]. The isolation of **6** from the Bermudan marine sponge *Leucetta microraphis* and its antimicrobial activity were initially reported by Kong and Faulkner [46], and the absolute configuration of **6** was elucidated by Dalisay et al*.* [47] using a deconvolution exciton coupled circular dichroism (CD) spectrum. In our study, compound **6** exhibited moderate antimycobacterial activity against *M. smegmatis* with an inhibition zone of 12 mm at 50 μg/disk, whereas its bis-TFA salt and *N,N’*-diacetyl derivative showed smaller inhibition zones, suggesting that the free amino groups in **6** are a key functional group for antimycobacterial activity. Although recent studies reported inhibitory effects on the Ubc13–Uev1A interaction and modulatory effects on TRPA1 and TRPM8 channels by **6** [48, 49], we were the first to demonstrate that compound **6** exhibited antimycobacterial activity.

Due to our continuous efforts, compound **2**, described in the previous section, was rediscovered as an anti-*M. smegmatis* substance with an MIC value of 16 μg/mL from two marine sponges *Halichondria panicea* and *Haliclona* sp. collected at Iriomote Island in Okinawa, Japan [39, 40]. With the isolation of **2** from Okinawan *Haliclona* sp., we also isolated new open-chain derivatives of **2**, namely, halichondriamine C (**7**) and 1-*epi*-halichondriamine C (**8**), as shown in Fig. 3, and reported their antimycobacterium activities against *M. smegmatis* with MIC values of 8.0 and 16 μg/mL, respectively [40]. Furthermore, alkaloids **7** and **8** both inhibited the growth of *M. bovis* BCG as a slow growing strain similar to *M. tuberculosis* with the same efficacy (MIC = 0.5 μg/mL for **7** and **8**), and were active against two more slowly growing mycobacterial strains, *M. avium* (MIC = 4.0 and 8.0 μg/mL for **7** and **8**, respectively) and *M. intracellulare* (MIC = 0.50 μg/mL for **7** and **8**), which are pathogens of *M. avium* complex (MAC) disease. MAC infection is an intractable pulmonary disease and its incidence has been increasing more than TB in developed countries. Anti-MAC drugs used clinically are limited and their therapeutic effects are insufficient [50, 51]. In our most recent study, we established an in vivo-mimic silkworm infection assay with MAC to efficiently screen anti-MAC antibiotics candidates with in vivo therapeutic efficacy [52] and, thus, a re-evaluation using this system is currently underway.

## Protein tyrosine phosphatase 1B and sterol *O*-acyl-transferase inhibitors

Lifestyle-related diseases, including type 2 diabetes mellitus (T2DM) and lipid metabolism disorders, are caused by unfavorable daily habits, such as a fat-rich diet, inadequate exercise, stress, and drinking/smoking, in addition to genetic factors and aging, and are now an increasing global issue [53, 54]. This section describes inhibitors of protein tyrosine phosphatase 1B (PTP1B) [55–58] and sterol *O*-acyl-transferase (SOAT, also known as acyl-CoA: cholesterol acyltransferase) [59–61], which are potential molecular targets for the treatment and prevention of these diseases.

PTP1B is expressed in the brain, liver, muscles, and adipose tissue and is a key negative regulator of the insulin signaling pathway [55]. Moreover, this enzyme has been shown to control the leptin signaling cascade [56], and, thus, the application of PTP1B inhibitors as anti-T2DM and obesity agents is expected [57, 58]. Since their clinical use has not yet been achieved despite a number of discoveries of natural and synthetic inhibitors [62–71], structurally novel types of drug candidates are in great demand.

**Fig. 4:**
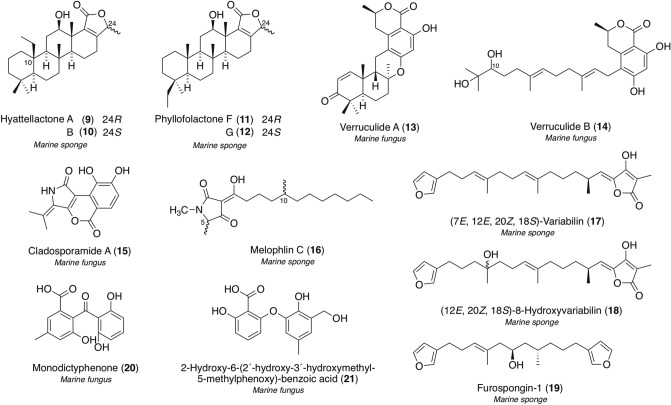
Structures of marine protein tyrosine phosphatase inhibitors **9**–**21**

An EtOH extract of the Indonesian marine sponge *Hyattella* sp. exhibited PTP1B inhibitory activity, and our bioactivity-guided separation led to the isolation of new hyattellactones A (**9**) and B (**10**), unique pentacyclic scalarane sesterterpenes possessing an α,β-unsaturated-γ-lactone ring and C-ethyl group [72], together with two known related sesterterpenes, phyllofolactones F (**11**) and G (**12**) (Fig. 4) [73]. Despite reports of more than 60 marine scalarane-type sesterterpenes with a C-ethyl group [74, 75], compounds **9** and **10** are the first examples to possess the ethyl group at the C-10 position. Compounds **9**/**10** and **11**/**12** are epimers at each C-24 position, and the 24*R*-isomers, **9** and **11**, exhibited more potent PTP1B inhibitory activity with IC_50_ values of 7.45 and 7.47 μM, respectively, than the 24*S*-isomers, **10** (42% inhibition at 24.2 μM) and **12** (inactive by 24.2 μM).

The fungal strain *Penicillium verruculosum* TPU1311 was separated from the ascidian *Polycarpa aurata*, and strong PTP1B inhibitory activity was observed in an extract of the culture broth. Using purification monitoring of its bioactivity, we isolated two new merosesquiterpenes, verruculides A (**13**) and B (**14**) (Fig. 4) [76], together with three known congeners, chrodrimanins A, B, and H [77–79]. Compound **14** had a linear sesquiterpene skeleton and may be a putative precursor before **13** is generated by the terpene cyclization reaction [80]. Although the absolute configuration of **14** at the C-10 position was not elucidated in our previous study, Gubiani and co-workers recently discovered 10*S*-**14** assigned by the in situ dimolybdenum CD method from the culture broth of *Phoma* sp. nov. LG0217 with an epigenetic modifier [81]. Compound **13** showed an IC_50_ value of 8.4 μM against PTP1B activity, while compound **14** exhibited reduced activity (40% inhibition at 23.1 μM), suggesting that the linear framework of **14** is not favorable for inhibitory activity.

A culture broth of the fungus *Cladosporium* sp. TPU1507, isolated from an unidentified marine sponge, exhibited PTP1B inhibitory activity, and the broth extract was fractionated with an ODS column and HPLC to give the new tricyclic metabolite with a 5/6/6 ring system, cladosporamide A (**15**) (Fig. 4) [82], as well as known prenylflavanone, (2*S*)-7,4′-dihydroxy-5-methoxy-8-(γ,γ-dimethylallyl)-flavanone [83, 84]. Compound **15** exhibited modest PTP1B inhibitory activity with an IC_50_ value of 48 μM.

Insulin and leptin signaling pathways are generally suppressed by other PTPs as well as PTP1B [55]. Among this family, the catalytic domains of T-cell PTP (TCPTP) and PTP1B share high homology; however, their biological functions markedly differ [85]. Accordingly, PTP1B/TCPTP selectivity is as important property, and TCPTP inhibitory activity by **15** was examined using an in vitro enzyme assay. Compound **15** inhibited TCPTP enzyme activity with an IC_50_ value of 54 μM; therefore, this compound is a dual inhibitor with equivalent potency against two PTPs, PTP1B and TCPTP. Previous studies using genetic techniques demonstrated that TCPTP knockout mice (*tcptp*^*–/–*^) had serious abnormalities [86, 87]; however, recent studies showed that knockout mice with a one-copy deletion of PTP1B and TCPTP *(ptp1B*^+*/–*^ or *tcptp*^+*/–*^) remained alive without any harmful phenotypes [88]. Therefore, the simultaneous inhibition of PTP1B and TCPTP has potential as a promising therapeutic strategy for T2DM and obesity.

In addition to the novel compounds described above, our successive studies afforded known compounds from Indonesian marine organisms as new types of PTP1B inhibitors.

Melophlin C (**16**), a known tetramic acid derivative, was isolated as the active constituent together with a new nortriterpenoid saponin, sarasinoside S, from the Indonesian marine sponge *Petrosia* sp. (Fig. 4) [89]. Compound **16** was initially obtained as a mixture of four diastereomers at the C-5 and C-10 positions from the Indonesian marine sponge *Melophlus sarassinorum* [90], and we also purified a similar isomeric mixture of **16**. However, the new saponin was inactive, whereas compound **16** inhibited PTP1B activity with an IC_50_ value of 14.6 μM and an inhibition of **16**-like tetramic acids was the first finding.

Three known furanoterpenes from two marine sponges, (7*E*, 12*E*, 20*Z*, 18*S*)-variabilin (**17**) [91–95] and (12*E*, 20*Z*, 18*S*)-8-hydroxyvariabilin (**18**) [94] from *Ircinia* sp. and furospongin-1 (**19**) [95] from *Spongia* sp., were discovered as unprecedented PTP1B inhibitors (Fig. 4) [96]. Compounds **17**–**19** exhibited PTP1B inhibitory activity with IC_50_ values of 1.5, 7.1, and 9.9 μM, respectively, and high cell viability. We previously identified the bicyclic furanoterpene, dehydroeuryspongin A as a new PTP1B inhibitor from the Okinawan marine sponge *Euryspongia* sp. [97, 98]: however, this was the first demonstration of linear-type furanoterpenes, such as **17**–**19**, inhibiting PTP1B activity. TCPTP inhibitory activities by **16** (IC_50_ of 0.8 μM versus 1.5 μM) and **17** (IC_50_ of 3.7 μM versus 7.1 μM) were approximately twofold as potent as that against PTP1B, whereas compound **19** showed equivalent IC_50_ values against TCPTP and PTP1B activities (9.6 μM versus 9.9 μM). Additionally, the selectivities of **17**–**19** over the other types of PTPs, CD45 tyrosine phosphatase (CD45 as a receptor-like PTP) and *vaccinia* H-1-related phosphatase (VHR as a dual-specificity phosphatase), were confirmed, suggesting that compound **17** exerted CD45 inhibitory effects (IC_50_ = 1.2 μM) similar to PTP1B, and its VHR inhibitory activity (IC_50_ = 6.0 μM) was four-fold less than that of PTP1B. Compound **18** non-selectively inhibited CD45 and VHR activities (IC_50_ = 9.0 and 9.4 μM, respectively), while compound **19** did not inhibit CD45 activity at 30 μM, but inhibited VHR activity with an IC_50_ value of 11 μM. These findings implied that the selective activities of the four PTPs were due to slight structural differences, carbon lengths, and modifications on **17**–**19**. Furanoterpenes are one of the major groups in marine sponge-derived natural products, and a number of derivatives have been reported [6, 7]. Therefore, further studies on structure–activity relationships and selectivities are our future plan.

Monodictyphenone (**20**), a known benzophenone derivative reported from a culture broth of the marine algicolous fungus *Monodictys putredinis* [99], was obtained along with the new biphenyl ether derivative, 2-hydroxy-6-(2′-hydroxy-3′-hydroxymethyl-5-methylphenoxy)-benzoic acid (**21**), by the fermentation of the fungus *P. albobiverticillium* TPU1432 isolated from an unidentified Indonesian ascidian (Fig. 4) [100]. PTP1B inhibitory activity in the broth was reproduced by **20** with an IC_50_ value of 36 μM. Compound **21** moderately exerted CD45 selective inhibitory effects (IC_50_ = 43 μM) among four PTPs, PTP1B, TCPTP, CD45, and VHR. CD45 as a receptor-like PTP critically controls lymphocyte signaling, and has recently been proposed as a promising drug target for autoimmune diseases [55].

SOAT, an endoplasmic reticulum membrane protein, catalyzes intracellular esterification, which transfers long-chain fatty acids generated by acyl-CoA to free cholesterol to biosynthesize the cholesteryl ester (CE) [59]. Therefore, this enzyme is a potential molecular target for the prevention of dyslipidemia, such as hypercholesterolemia and related diseases, caused by the excessive accumulation of CE [60, 61]. Moreover, recent molecular biology studies revealed that SOAT has two SOAT isozymes, SOAT1 and SOAT2, the localization and functions of which markedly differ [59]. Since the selectivities of SOAT1 and SOAT2 are considered to be an important index [101], we have been evaluating SOAT inhibitory activity toward these two isozymes using African Green monkey-derived SOAT1 and SOAT2 gene-expressing CHO cells (SOAT1-CHO and SOAT2-CHO cells) [102–107].

**Fig. 5:**
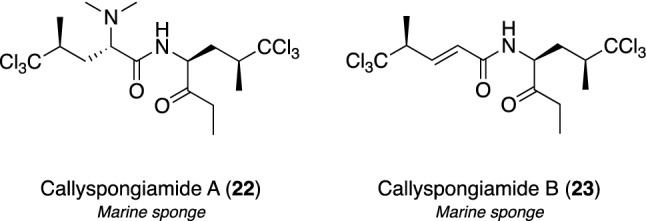
Structures of callyspongiamides A (**22**) and B (**23**) as SOAT inhibitors

The screening study on SOAT1/SOAT2 inhibitors afforded an EtOH extract of the Indonesian marine sponge *Callyspongia* sp., and the separation process provided two new polychlorine-containing modified dipeptides, callyspongiamides A (**22**) and B (**23**) (Fig. 5) [108], together with the known congener, dysamide A [109]. The effects of **22** and **23** on the synthesis of CE through the inhibition of SOAT1/SOAT2 isozymes were examined using SOAT1-CHO and SOAT2-CHO cell-based assays and the respective IC_50_ values over SOAT1 and SOAT2 were 0.78 and 2.8 μM for **22** and 1.2 and 2.4 μM for **23**, respectively. To identify the molecules of **22** and **23** inhibiting the accumulation of CE, their inhibitory activities against SOAT1/SOAT2 isozymes were also examined using an enzyme assay with microsomes prepared from SOAT1-CHO and SOAT2-CHO cells, respectively. Compounds **22** and **23** affected SOAT1/SOAT2 enzyme activities with IC_50_ values of 0.23/0.86 μM for **22** and 1.0/3.2 μM for **23**, respectively, which were similar to inhibitory activities in the cell-based assay. Based on these findings, compounds **22** and **23** are dual-type SOAT1 and SOAT2 inhibitors.

**Fig. 6:**
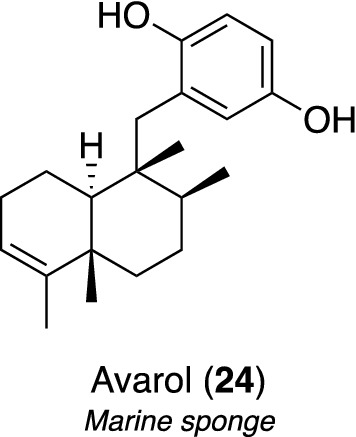
Structure of avarol (**24**) as a multifunctional inhibitor against PTP1B and SOAT1/2

We recently reported marine sesquiterpene hydroquinones, including three new derivatives, avapyran, 17-*O*-acetylavarol, and 17-*O*-acetylneoavarol, from the marine sponge *Dysidea* sp. collected at Iriomote Island (Okinawa, Japan) [110]. Of these, avarol (**24**), which was initially isolated from the marine sponge *Disidea avara* [111], was identified as be a multifunctional inhibitor of PTP1B and SOAT1/2 (Fig. 6) [110, 112]. Compound **24** had an IC_50_ value of 12 μM against PTP1B and blocked CE synthesis by inhibiting SOAT1/SOAT2 isozymes in SOAT1-CHO and SOAT2-CHO cells with IC_50_ values of 14.2 and 14.8 μM, respectively. These findings proposed compound **24** as a multitarget-directed lead compound for the attenuation of metabolic syndromes.

## Inhibitors of BMP-induced osteoblastic differentiation

Bone morphogenetic protein (BMP), a member of the transforming growth factor-β superfamily, plays an important role in the formation and repair of bone [113, 114]. Therefore, the disruption of BMP signaling causes several types of bone disorders.

Fibrodysplasia ossificans progressiva (FOP) is a rare congenital disorder caused by abnormal BMP signaling activated by a mutant BMP receptor [activin receptor-like kinase-2 (ALK2)], leading to progressive heterotopic ossification (HO) in soft tissues [115, 116]. Since BMP signaling inhibitors are a promising strategy for the prevention of HO [117, 118], we started a screening program by monitoring the BMP-induced osteoblastic differentiation of a C2C12 cell line stably expressing mutated ALK2(R206H) (C2C12(R206H) cells) [119, 120].

**Fig. 7:**
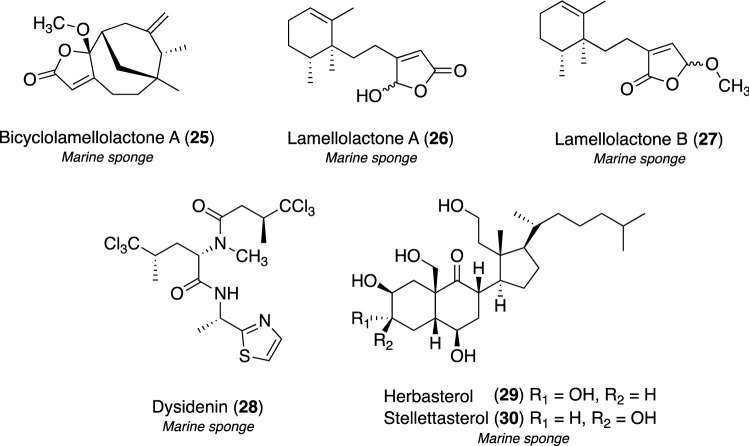
Structures of marine osteoblastogenesis inhibitors **25**–**30**

Approximately 200 Indonesian marine invertebrates, marine sponges and ascidians, have been screened using the C2C12(R206H) cell-based assay, and an EtOH extract of the marine sponge *Lamellodysidea* sp. (cf. *L. herbacea*) was found to inhibit the BMP-induced osteoblastic differentiation of C2C12(R206H) cells [121]. Bicyclolamellolactone A (**25**), a new sesquiterpene lactone with an unusual bicyclo[4.3.1]decane ring, was isolated together with two monocyclofarnesol-type sesquiterpenes, lamellolactones A (**26**) and B (**27**) [122], through bioactivity-guided purification (Fig. 7). The planar structure of **25** was elucidated based on spectroscopic data, including 1D and 2D NMR spectra. The stereoconfiguration of **25** was completely assigned by the calculation of electric CD (ECD) spectra and NOESY correlations. Compounds **25**–**27** inhibited the BMP-induced osteoblastic differentiation of C2C12(R206H) cells with IC_50_ values of 51, 4.6, and 20 μM, respectively, and no cytotoxic effects.

We originally discovered compounds **26** and **27** from another *Lamellodysidea* sp. marine sponge collected in Indonesia; however, their biological activities were not identified in a previous study [122]. Our sustained efforts enabled the rediscovery of **26** and **27** as BMP-induced osteoblastogenesis inhibitors.

Among the samples screened, an EtOH extract of the marine sponge *Dysidea* sp. also exerted potent inhibitory effects on osteoblastogenesis, and repeated column purification based on this activity led to the isolation of three active constituents [123]: dysidenin (**28**) [124–126], herbasterol (**29**) [127], and stellettasterol (**30**) (Fig. 7) [128]. The inhibitory effects of **28**–**30** on the BMP-induced osteoblastic differentiation of C2C12(R206H) cells showed IC_50_ values of 2.3, 4.3, and 4.2 μM, respectively, with no cytotoxicity. Since the BMP signaling pathway is transduced through the transcriptional factors Smad1/5 [116], a BMP-Smad-specific Id1WT4F-luciferase reporter assay was performed to examine the direct effects of **28**–**30** on cell signaling [129]. This reporter assay revealed that no compounds inhibited luciferase activity by 18.4–21.4 μM, indicating that the molecular targets of **28**–**30** are downstream of the Smad transcriptional step in the BMP signaling cascade.

Our collaborative research covers terrestrial resources, and phytochemical studies have also been conducted to screen bioactive constituents from the Indonesian medicinal plants, *Wedelia prostrata*, *Lantana camara*, *Rhinacanthus nasutus*, *Spilanthes paniculata*, and *Syzygium polyanthum* [130–136]. If there is another opportunity, the details of these compounds will be reviewed elsewhere.

## Marine-derived organohalides

We have demonstrated that marine environments offer a structurally and biologically diverse range of natural products [6, 7]. Additionally, organisms living in the sea, including marine sponges, ascidians, microorganisms, cyanobacteria, algae, and mollusks, are a rich source of organohalides [6, 7, 137–139]. Halogenated natural products have been reported to exhibit various biological activities [6, 7, 140], and, for example, vancomycin as a clinical antibiotic is mainly used to treat methicillin‐resistant *Staphylococcus aureus* (MRSA) infection [141].

**Fig. 8:**
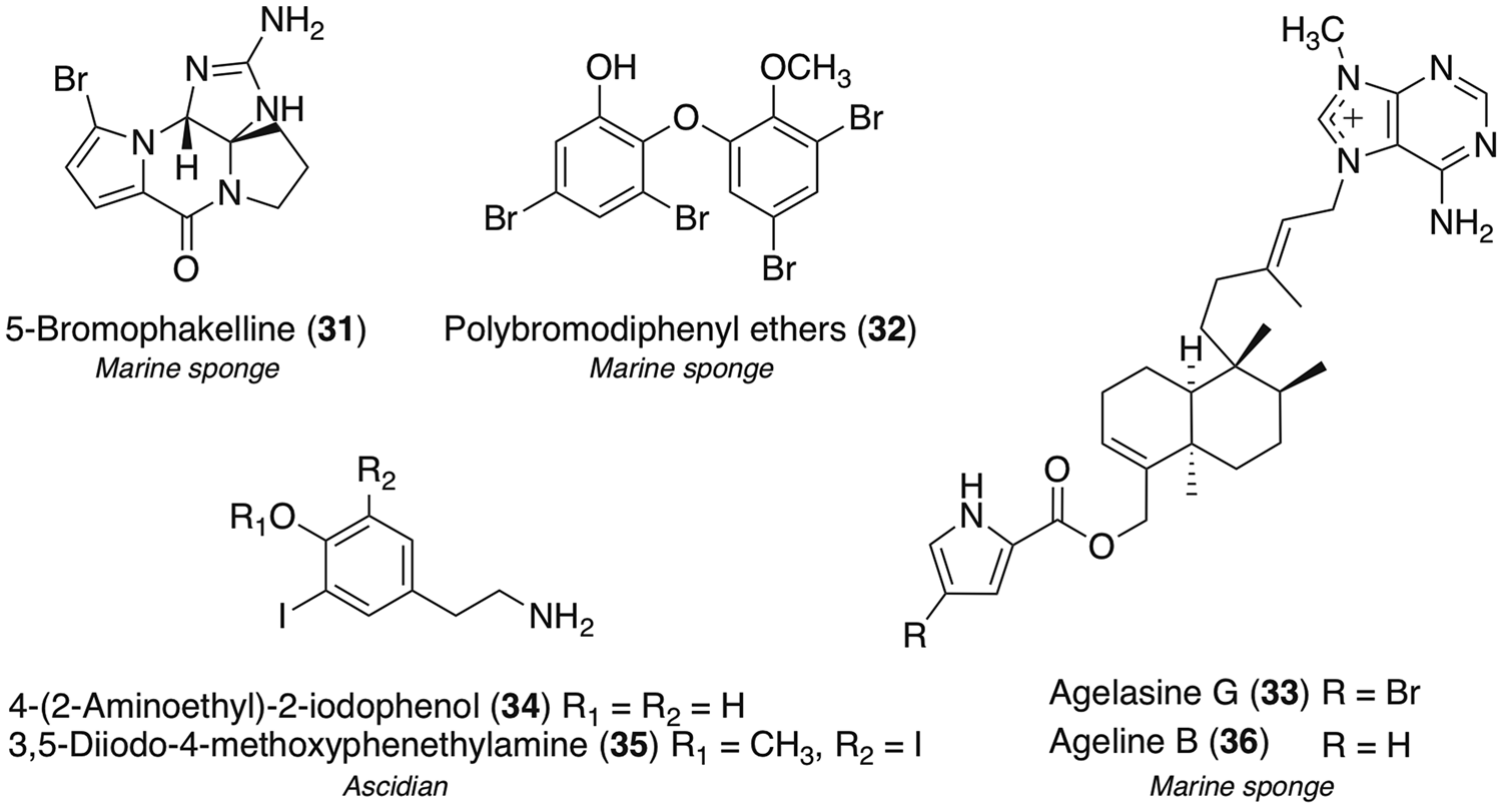
Structures of marine organohalogens **31**–**35** and related compound **36**

In the course of our screening study on marine resources, bromopyrrole alkaloids along with the new analog, 5-bromophakelline (**31**), from the Indonesian marine sponge *Agelas* sp. [38], some known polybromodiphenyl ethers (**32**) from two Indonesian marine sponges *Lamellodysidea* spp. [122, 142], agelasine G (**33**), a known bromo-containing diterpene with *N*-methyladenine, from the Okinawan marine sponge *Agelas nakamurai* [143], and known tyramine derivatives with rare iodine groups, 4-(2-aminoethyl)-2-iodophenol (**34**) and 3,5-diiodo-4-methoxyphenethylamine (**35**), from an Indonesian assidian *Didemnum* sp. [144] have been isolated in addition to the chlorinated compounds **22**, **23**, and **28** (Fig. 8). Of these marine organohalogens, we herein introduce compound **33** with unique biological properties [145].

Agelasine G (**33**), which belongs to a large group of marine natural products, was originally isolated from the Okinawan marine sponge *Agelas* sp. by Kobayashi and co-workers in 1992 and its structure comprises bromopyrrole, *N*-methyladeninium, and diterpene moieties [146]. In the process of screening anti-*M*. *smegmatis* substances from the marine sponge *A. nakamurai* collected at Iriomote Island (Okinawa, Japan), we isolated new antimycobacterial agelasine derivatives and discovered PTP1B inhibitory activity by **33** with an IC_50_ value of 15 μM for the first time, while ageline B (**36**) [147], a known debromo-derivative of **33** obtained from the same marine sponge, was inactive up to 19 μM. These findings indicated that a Br atom is responsible for the inhibition of PTP1B activity, which is supported by our previous findings showing that polybromodiphenyl ethers exhibited more potent PTP1B inhibitory activity than diphenyl ether derivatives without Br groups [100, 122, 142, 148]. As described in the section on PTP1B inhibitors, PTPs are composed of 107 members, including PTP1B as non-transmembrane PTPs, and regulate various cellular functions [55]. The inhibitory effects of **33** and **36** toward three types of PTPs, TCPTP, CD4, and VHR, were evaluated using an in vitro enzyme assay. Compound **33** was only active against VHR (IC_50_ = 13 μM) with a similar potency to that against PTP1B, while compound **36** did not affect any PTPs by 19 μM.

**Fig. 9:**
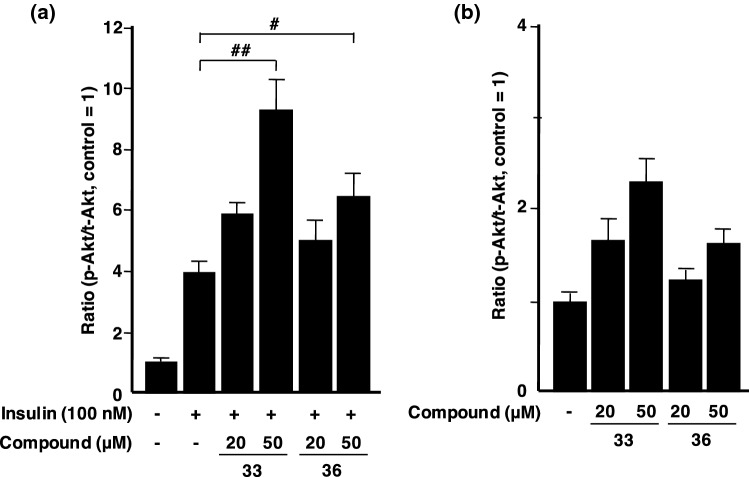
**a** Enhanced effects of agelasine G (**33**) and ageline B (**36**) on insulin-stimulated Akt phosphorylation levels in Huh-7 cells. **b** Effects of **33** and **36** on Akt phosphorylation levels in Huh-7 cells. p-Akt/t-Akt levels were shown as a ratio of that in the control group. Data are expressed as the mean ± SE (*n* = 4). ^#^*P* < 0.05, ^##^*P* < 0.01 vs the insulin treatment group

To demonstrate their cellular effects, the phosphorylation levels of Akt (p-Akt), a key downstream molecule of the insulin signaling pathway starting from the insulin receptor, were measured by Western blotting using human hepatoma Huh-7 cells, in which PTP1B is mainly located. In this assay, compound **33** increased insulin-stimulated p-Akt levels in Huh-7 cells in a dose-dependent manner, suggesting that the inhibition of PTP1B activity by **33** activates the insulin signaling pathway. On the other hand, compound **36**, an inactive derivative, also moderately enhanced insulin-stimulated p-Akt levels in a dose-dependent manner (Fig. 9a). These findings implied that compounds **33** and **36** have additional target molecule(s) that activate the cascade besides the inhibitory effects of PTP1B activity. Therefore, the effects of **33** or **36** alone on the p-Akt level of the signaling pathway in Huh-7 cells were tested using the same experiments without the insulin stimulation. Although compounds **33** and **36** did not significantly increase p-Akt levels at 50 μM, slight dose-dependent elevations in p-Akt levels were detected in Huh-7 cells (Fig. 9b). These findings suggest that compounds **33** and **36** exert insulin-like effects to activate insulin signaling at an upstream point instead of insulin.

Therefore, compounds **33** and **36** initially exert similar effects to insulin for signal transduction, and compound **33** inhibited PTP1B activity to activate downstream of the signaling pathway. Considering these compounds in terms of their chemical structures, the presence of a Br group is significant for the inhibition of PTP1B, while the terpene and/or adenine moieties may contribute to insulin-like effects. Many types of PTP1B inhibitors have been obtained from natural and synthetic origins; however, clinically efficient drugs have not yet been developed [57, 58, 62]. PTP1B inhibitors with insulin-like activity are extremely rare, and, thus, we are now investigating the optimal structures for these biological properties with the aim of developing candidate agents for the treatment of T2DM and obesity.

## Efficient production of halogenated metabolites by fungal strains

From the above achievements, we were further interested in halogenated natural products that exhibit significant biological activity. Therefore, we attempted fermentation study with a focus on fungal strains to efficiently produce halogen-containing metabolites [149–155].

**Fig. 10:**
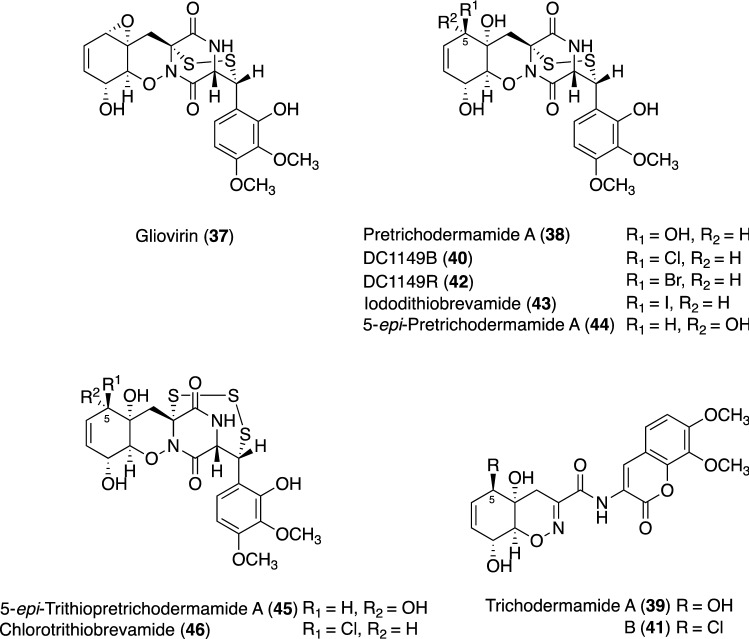
Structures of **37**–**46** from a culture broth of the Palauan marine-derived fungus *Trichoderma* sp. TPU199

In our trials, the Palauan marine-derived fungus *Trichoderma* sp. TPU199 (cf. *T. brevicompactum*) from an unidentified red alga was found to possess objective productivity [151]. Under ordinary culture conditions using freshwater in our laboratory, this fungal strain produced the unique metabolites, gliovirin (**37**) [156, 157], pretrichodermamide A (**38**) [158], and trichodermamide A (**39**) (Fig. 10) [159]. Although compounds **37** and **38** were generally categorized into the epipolythiodiketopiperazine (ETP) family (also known as epipolythiodioxopiperazine), cyclic dipeptides with a sulfide bridge (–S–, –SS–, –SSS–, or –SSSS–) between the α-positions of two amino acid residues [160], ETPs **37** and **38** formed an unprecedented disulfide linkage between the α- and β-positions of two amino acids (called gliovirin-type ETP in our study [151]).

Since strain TPU199 is a marine-derived fungus, the next fermentation was performed using sterilized natural seawater medium. This condition reduced the production of **37** and newly gave two peaks (**40** and **41**) in a seawater concentration-dependent manner. The structures of **40** and **41** isolated under the seawater condition were elucidated as the chlorinated derivatives of **38** and **39**, DC1149B [161] and trichodermamide B [159], respectively, based on their spectroscopic data (Fig. 10), inferring that the Cl groups of **40** and **41** were incorporated from NaCl in the seawater medium. Subsequent conditions using 3.0% NaCl- or NaBr-supplemented medium were examined and led to the production of known halogenated gliovirin-type ETPs possessing the Cl and Br groups, DC1149B (**40**) and DC1149R (**42**), respectively (Fig. 10) [161].

Compound **42**, a brominated derivative of **38**, was documented in the same patent as **40** and was semisynthetically obtained from **37** by a reaction with HBr; however, the ^1^H and ^13^C NMR assignments of **42** have not been reported [161]. Therefore, we were the first to describe the isolation of **42** as a fungal fermentation product as well as the complete assignment of ^1^H and ^13^C NMR spectroscopic data for **42** [150].

NaI was supplemented into the culture medium in anticipation of the production of iodinated metabolites, and the HPLC chromatogram of the broth with 3.0% NaI displayed a new peak, corresponding to metabolite **43**, with similar UV spectrum to those of **37**, **38**, **40**, and **42**. Newly emerging **43** was purified by an ODS column and HPLC from the broth extract and 1D and 2D NMR analyses revealed the structure of **43** to be a new iodinated derivative of **38**, named iododithiobrevamide (Fig. 10) [151]. Various bromine-added metabolites were previously reported to be generated by fermentation with inorganic bromides [162–164]; however, obtaining the I derivative using the fermentation method with NaI is a rare and interesting finding.

Our precise purification of strain TPU199 on the NaI-containing culture broth more recently resulted in the isolation of two new gliovirin-type ETPs **44** and **45** [151], which were elucidated as 5-*epi*- and 5-*epi*-trithio-**38**, respectively (Fig. 10). An *N*-methyl derivative of **44**, designed as pretrichodermamide F, was initially reported as a gliovirin-type ETP with a 5α-oriented substituent [165], and, thus, our findings are the second documented report of these ETPs. Pretrichodermamide derivatives with the 5α-oriented substituent, **44** and **45**, may be generated via nucleophilic substitution from iodinated gliovirin-type ETP, such as **43**. NaI-supplemented cultivations represent a versatile method to yield structurally diversified metabolites, not only the production of iodinated metabolites, but also stereoisomers.

Furthermore, in the course of investigations on culture conditions, a seawater culture of the TPU199 strain with 1.0% dimethyl sulfoxide (DMSO) provided a new gliovirin-type ETP named chlorotrithiobrevamide (**46**), the structure of which was confirmed to be a trithio-derivative of **40** (Fig. 10). In contrast to this condition, the production of **46** was not detected by the addition of DMSO to a freshwater medium [152]. Compound **46** was the first example of a trithio-derivative in the gliovirin-type ETP, and recent studies added outovirin C and penicisulfuranol C produced by *P. raciborskii* TRT59 and *P. janthinellum* HDN13-309, respectively, as the second and third examples followed by **45** in this series of ETPs [150, 166, 167].

**Fig. 11:**
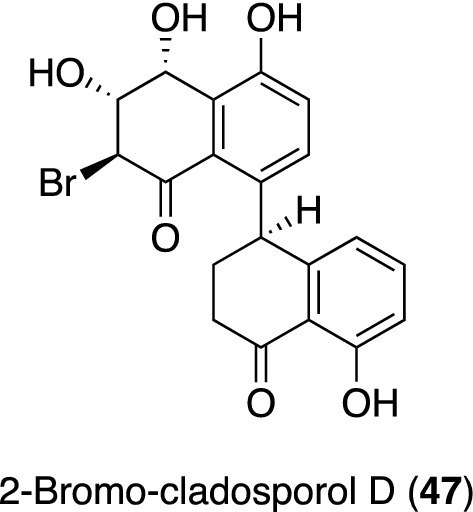
Structure of 2-bromo-cladosporol D (**47**) from a culture broth of the Okinawan plant-associated fungus *Cladosporium* sp. TMPU1621

The fungal strain *Cladosporium* sp. TMPU1621 isolated from the leaves of Okinawan *Achyranthes aspera* var. *rubrofusca* was identified as the second producer with the productivity of organohalides [155]. The TMPU1621 strain produced a series of cladosporol derivatives [168–170], including a chlorinated congener under freshwater medium conditions, and the supplementation of 3.0% NaCl into the medium increased the production of chlorinated cladosporol. Therefore, we examined 3.0% NaBr-supplemented medium to induce the production of a new brominated derivative, and, as expected, obtained 2-bromo-cladosporol D (**47**) (Fig. 11). Compound **47** exhibited modest anti-MRSA activity with an MIC value of 25 μM, whereas the chlorinated congener was inactive by 50 μM. However, iodinated cladosporols have not yet been isolated from the culture broth of the strain with NaI-containing medium. Since its HPLC chromatogram differs from those obtained under other culture conditions, further studies are warranted (unpublished data).

Although these culture methods are very simple and easy, the probability of discovering objective strains with the desired characteristics is still low due to the extremely poor growth of microorganisms in medium containing halide salts or DMSO. Therefore, our aim is to develop novel strategic approaches to produce halogenated microbial metabolites, and the data obtained will be published in the near future.

## Conclusion

Countless bioactive products have historically been reported from plants and microbes, and notable examples, such as paclitaxel (antineoplastic), artemisinin (antimalarial drug), penicillin (antibiotic), lovastatin (antihyperlipidemic agent), tacrolimus (immune suppressant), and ivermectin (antiparasitic), have contributed to breakthroughs in modern medicine. However, it has become increasingly difficult to identify novel natural products with excellent biological activities, and, thus, unutilized natural resources (ocean or extreme environments) and innovative searching strategies are required to expand this research field.

We herein described various Indonesian marine biological substances obtained by a collaborative screening program with UNSRAT. Marine natural product chemistry has rapidly advanced in a short period of time and has already provided several clinical agents. Therefore, we expect our findings to serve as drug seed/lead compounds for clinical applications to cancer, TB, T2DM, dyslipidemia, and FOP. This review also described the induced production of fungal organohalogens on which we started to work with inspiration based on the unique biological activities of marine halogenated products. The present methods may not be straightforward strategies, but simple techniques would enable researchers in chemical laboratories to access new compounds. These findings will facilitate and accelerate drug discovery and development in natural product chemistry.
